# Apoferritin encapsulation of cysteine protease inhibitors for cathepsin L inhibition in cancer cells†

**DOI:** 10.1039/c9ra07161j

**Published:** 2019-11-11

**Authors:** José C. Quilles Junior, Fernanda dos Reis Rocho Carlos, A. Montanari, Andrei Leitão, Viviane W. Mignone, Maria Augusta Arruda, Lyudmila Turyanska, Tracey D. Bradshaw

**Affiliations:** Centre for Biomolecular Sciences, School of Pharmacy, University of Nottingham NG7 2RD UK quilles@usp.br tracey.bradshaw@nottingham.ac.uk; Medicinal Chemistry Group (NEQUIMED), São Carlos Institute of Chemistry (IQSC), University of São Paulo Av. Trabalhador São-carlense, 400, São Carlos SP 13.566-590 Brazil; Queen's Medical Centre, School of Life Sciences, University of Nottingham NG7 2RD UK; School of Physics and Astronomy, University of Nottingham NG7 2RD UK lyudmila.turyanska@nottingham.ac.uk

## Abstract

Cysteine proteases play a key role in tumorigenesis causing protein degradation and promoting invasive tumour growth. Cathepsin L is overexpressed in cancer cells and could provide a specific target for delivery of anticancer agents. We encapsulated novel dipeptidyl nitrile based cysteine protease inhibitors (Neq0551, Neq0554 and Neq0568) into biocompatible apoferritin (AFt) protein nanocages to achieve specific delivery to tumours and pH-induced drug release. AFt-encapsulated Neq0554 demonstrated ∼3-fold enhanced *in vitro* activity (GI_50_ = 79 μM) compared to naked agent against MiaPaCa-2 pancreatic carcinoma cells. Selectivity for cancer cells was confirmed by comparing their activity to non-tumourigenic human fibroblasts (GI_50_ > 200 μM). Transferrin receptor (TfR-1) expression, detected only in lysates prepared from carcinoma cells, may contribute to the cancer-selectivity. The G_1_ cell cycle arrest caused by AFt-Neq0554 resulting in cytostasis was corroborated by clonogenic assays. Superior and more persistent inhibition of cathepsin L up to 80% was achieved with AFt-encapsulated agent in HCT-116 cells following 6 h exposure to 50 μM agent. The selective anticancer activity of AFt-encapsulated cysteine protease inhibitor Neq0554 reported here warrants further preclinical *in vivo* evaluation.

## Abstract 2

Novel apoferritin encapsulated cysteine protease inhibitors are developed with enhanced and selective uptake by cancer cells, and sustained pH-induced release of the agent. The persistent inhibition of cathepsin L is demonstrated *in vitro*.

## Graphical abstract

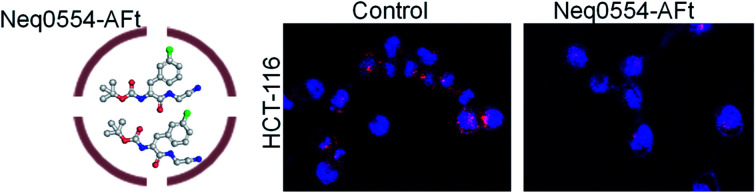

## Introduction

Proteases are essential for cell survival and development and play important roles in cellular proliferation, migration, adhesion, senescence, autophagy, apoptosis and immune system evasion.^2^ To date, proteases were considered as potential targets for the treatment of diseases, such as Chagas disease,^3^ leishmaniosis,^4^ osteoporosis^5^ and some types of carcinomas.^6–8^ Predominantly found in the intracellular environment, these enzymes are abundantly localized in the lysosomes of normal cells.^9^ However, some proteases are also present in the extracellular medium, assisting extracellular matrix degradation in cancer.^2,10^

Cathepsin L, a lysosomal cysteine protease, is a marker for, and overexpressed in pancreatic cancer.^11,12^ Because of the aggressive nature of pancreatic cancers, >80% of patients present with metastatic disease and 5 years survival is dismal, ∼8%.^10,13^ Colorectal adenocarcinoma (CRC) also represents an aggressive, chemotherapy-resistant disease.^14^ With high metastatic incidence (>50% patients) and poor prognosis associated with late stage disease (5 years survival stage IV CRC < 8%), therapeutic intervention has limited success.^14^ Cellular deregulation of cathepsin L expression is one of the common characteristics of these types of carcinomas, as well as elevated extracellular levels.^15,16^ Inhibition of cathepsin L could offer a route for treatment of these cancers. Recently, elevated cathepsin L activity has been reported in murine models with pancreatic cancer, with reduction in the tumour size after its gene deletion.^13^ Cathepsin L inhibition by covalent inhibitors was shown to suppress proliferation of pancreatic cancer cells,^11^ and colorectal adenocarcinoma cells.^14^

Reversible covalent inhibitors are known to be a viable approach to decrease side effects associated with the off-target effects inside the cells.^17^ However, all types of covalent inhibitors need to position the reactive group in the vicinity of the cysteine amino acid in the catalytic pocket, thus leading to the necessity to design compounds with substituents that fit the subsites of the enzyme counterpart mimicking a peptide (peptidomimetics). One of the promising groups of peptidomimetic agents are dipeptidyl nitrile reversible covalent cysteine protease inhibitors with high-affinity for the target enzyme and potent human cathepsin L and cruzipain inhibition efficiency.^18,19^ Cathepsin L possesses important functions in normal cells, and its inhibition could cause adverse toxicities limiting therapeutic efficacy. Hence new formulations are needed to ensure selective or preferential uptake by cancer cells.

The apoferritin (AFt) nanocapsule has been identified as an ideal drug delivery vehicle^20^ and has been used to encapsulate proteins and small drug molecules.^21–24^ AFt is internalized into cells by transferrin receptor 1 (TfR1)-mediated endocytosis; TfR1 is upregulated and highly expressed on cancer cell membranes.^25^ Also, enhanced delivery and uptake to cancer tissues is expected due to enhanced permeability and retention (EPR) associated with the tumour microenvironment.^26^ In the cells, AFt is trafficked to lysosomes, where increased acidity will increase the protein pore size releasing the cargo.^24^ Since cathepsin L is found predominately within lysosomes, AFt could offer great potential for selective delivery of cysteine protease inhibitors.

Here we report the development of a new formulation of dipeptidyl nitrile derivatives for selective targeting to cancer cells and pH dependent drug release. We encapsulated cysteine protease inhibitors (Neq0551, Neq0554 and Neq0568) within AFt protein cages, demonstrating retention of AFt capsule structural integrity and formulation stability. Investigation of AFt formulations in physiologically relevant conditions revealed enhanced drug release under acidic pH 5.5, associated with tumour microenvironments, compared to neutral pH 7.4. *In vitro* assessment of antitumor activity of naked and AFt-encapsulated agents against pancreatic and colorectal cancer cells was performed, confirming cancer-selectivity and enhanced potency of the developed formulation of these inhibitors. Our results provide the first demonstration of the potential of AFt for targeted delivery of cysteine protease inhibitors to cancer cells, relevant for their applications as anticancer agents in clinic.

## Materials and methods

### Preparation and characterization of encapsulated compounds

All the covalent reversible cysteine protease inhibitors were synthesized as previously reported.^18^ Agents, with purity >95% according to HPLC-MS analysis, were selected based on their biochemical potential to inhibit cysteine proteases in the nanomolar range. Horse spleen AFt was diluted in sodium acetate buffer 100 mM pH 5.5 at 6 × 10^−9^ as final numbers of moles and kept at 4 °C. Every 45 min, the drugs dissolved in dimethylsulfoxide (DMSO) at the stock concentration of 50 mM were gently added into the AFt solution to give a final ratio of 1 : 500 (AFt : drugs). Un-encapsulated compound was removed from solution by ultracentrifugation using Amicon membranes 30 kDa (13 000 rpm, 4 min, 4 °C). Neq compound concentration after purification of AFt-encapsulated compound was determined by UV-Vis absorbance at 248 nm using a Thermo Fisher Scientific NanoDrop™ 2000/c Spectrophotometer; the Beer–Lambert law was used to quantify encapsulation. Total protein concentration was determined using the Bradford assay.^27^ Encapsulation efficiency (EE%) is calculated as the percentage of drug that is successfully entrapped into the apoferritin with respect to the drug added. Drug loading (DL%) is calculated as the amount of drug loaded with respect to the total weight of the nanoparticle (apoferritin and encapsulated drug molecules).

All encapsulated drugs were aliquoted and stored at 4 °C pH 5.5 and stability was examined throughout 6 weeks by UV-Vis spectroscopy. The physical properties of AFt were characterized by DLS and zeta-potential (using 1 mL of 0.2 mg mL^−1^ AFt-encapsulated agent). Native PAGE was conducted to confirm AFt protein structure after encapsulation. Proteins, 15 μL AFt solutions (∼0.2 mg mL^−1^) were separated on a 4–16% gradient gel (Novex) at 4 °C using cathodic and anodic buffers. Proteins were stained following immersion of gels in Coomassie brilliant blue for 1 h and washed with deionized water before capture of images using Gene flow limited. Drug release was analyzed *in vitro* in acidic (sodium acetate 100 mM pH 5.5) and neutral (Hepes buffer 100 mM pH 7.4) pH conditions. A dialysis method was selected using a dialysis membrane (cut-off 8 kDa) for 24 h at 37 °C. At different time points, drug release was determined by UV-Vis, considering the initial concentration of encapsulated drug as 100%.

### *In vitro* studies

HCT-116 (hMLH1-) colorectal carcinoma cells and MiaPaCa-2 pancreatic adenocarcinoma cells were purchased from the American type culture collection (ATTC) and grown in RPMI-1640 medium supplemented with 10% foetal bovine serum (FBS). For MRC-5 human fibroblast cells, DMEM medium modified with 1% antibiotics, 1% non-essential amino acids, 5 mM l-glutamine, 1 mM HEPES buffer and 10% FBS was used. All cell lines were grown at 37 °C, 5% CO_2_ and used for the assays after achieving 70% confluence. The MTT [3-(4,5-dimethylthiazol-2-yl)-2,5-diphenyltetrazolium bromide] assay was used for cell growth and viability determination of normal and cancer cell lines after treatment with naked and encapsulated drugs at different concentrations. Cells were seeded into 96-well plates at density of 3 × 10^3^ per well. The MTT reduction at the time of drugs' addition (*T*_0_) and following 72 h exposure was assessed to determine growth inhibitory effects.

The clonogenic cell survival test was adopted to determine the ability of single cells to survive a brief exposure to test agents and maintain proliferative potential to form progeny colonies. For this study, the cells (300 per well) were seeded into 6-well plates and test agent treatment was performed for 24 h using naked and encapsulated compounds at 10 μM and 100 μM. After 7 days of incubation, colonies were stained with 0.5% methylene blue.

### Cell cycle study

For cell cycle analysis cell were seeded in 6 well plates at a seeding density of 5 × 10^5^ cells per well in 2 mL medium. Following treatment with naked and encapsulated Neq0554 at final concentration of 10 μM and 100 μM, cells were pelleted by centrifugation then resuspended in 0.5 mL fluorochrome solution 50 μg mL^−1^ propidium iodide (PI), 0.1 mg mL^−1^ ribonuclease A, 0.1% v/v Triton X-100, and 0.1% w/v sodium citrate in deionized water (dH_2_O). Cell suspensions were kept overnight at 4 °C protected from light. Cell cycle analyses were performed on a Beckman Coulter FC500 flow cytometer. EX-PO32 software was used to analyze data.

### Enzymatic studies

Cathepsin L isolated from human liver (Enzo life sciences) was assayed fluorometrically using the system Biotek Synergy HT at 25 °C with a fluorescence emission of 460 nm (excitation of 355 nm) over 5 minutes. The hydrolysis rate of the fluorogenic substrate Z-Phe-Arg-7-amido-4-methylcoumarin (Z-FR-MCA, Sigma-Aldrich) was monitored. Enzyme kinetic assays were carried out in Corning 96-well black flat bottom microplates containing 200 μL of a solution constituted by 100 mM acetate buffer pH 5.5, 300 mM NaCl, 7 mM DTT (dithiothreitol), 5% v/v DMSO, 0.01% v/v TritonX-100 and 1.9 nM hCat-L. The enzyme was activated for 20 min in the assay buffer (100 mM acetate pH 5.5 and 7 mM DTT) before the reaction started. The substrate Z-FR-MCA was prepared with a final concentration of 10.9 μM (=4 × *K*_M_). Stock solutions of the inhibitors were prepared in DMSO with initial concentrations varying from 1 to 10 μM. The assay was performed in triplicate. Analysis and manipulation of the data were performed with Origin Pro 8.5. Each experiment was performed in triplicate for each substance. Initial velocities of the substrate hydrolysis under the first-order reaction were calculated using Gen5™ Biotek software. The apparent inhibition constant 
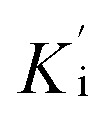
 was determined by non-linear regression using equation 
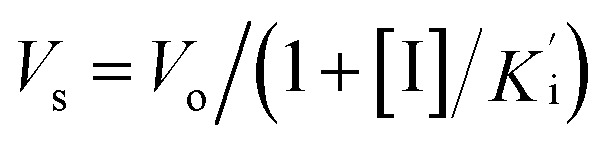
, where *V*_s_ is the steady-state rate, *V*_o_ is the rate in the absence of inhibitor, and [I] is the inhibitor concentration. The true inhibition constant *K*_i_ was calculated by the correction of 
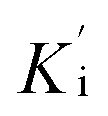
 according to 
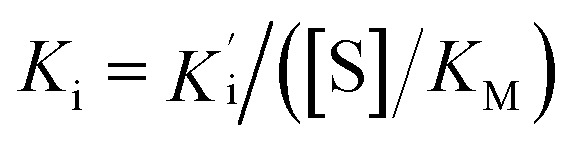
, where [S] is the substrate concentration and *K*_M_ is the Michaelis constant.

### Western blot

Cells were cultured in 75 cm^2^ flasks at 37 °C, 5% CO_2_ until ∼70% confluent. Cells were then detached by trypsin and lysed using lysis buffer (100 mL NP40, 1 M NaCl, 1 M Tris–HCl pH 8.0) supplemented with a protease inhibitor cocktail (Roche). Cellular proteins (50 μg) were separated by sodium dodecyl sulfate-polyacrylamide gel electrophoresis (SDS-PAGE), and electro-transferred onto PVDF membranes blocked in Tris-buffered saline (TBS) solution containing 5% milk at room temperature. Membranes were incubated in primary antibodies (1° Abs; GAPDH and TfR-1) overnight at 4 °C. Membranes were then washed with TBS solution at room temperature and incubated with a secondary (2°) Ab (GE) for 1 h. Detection was performed with Super Signal chemiluminescent reagent according to the manufacturer's protocol (Tanon, China).

### Confocal fluorescence microscopy

Cells were seeded into 96-well black plates at a density of 10^4^ cells per well and treated with 100 μM of naked and encapsulated compounds for 24 h. Cells were washed with PBS and incubated for 1 h with 300× diluted Red Magic Substrate (Sigma-Aldrich) and Hoechst as a nuclear marker. Fluorescence images were acquired with a confocal microscope Ultra Nikon equipped with a 40× objective with excitation filters at 510–560 nm. Fluorescence emission was detected using bandpass filter at 570–620 nm. Non-treated cells and cells treated with AFt alone were used as negative controls.

## Results

### Encapsulation and characterization of AFt-Neq test agents

**Fig. 1:**
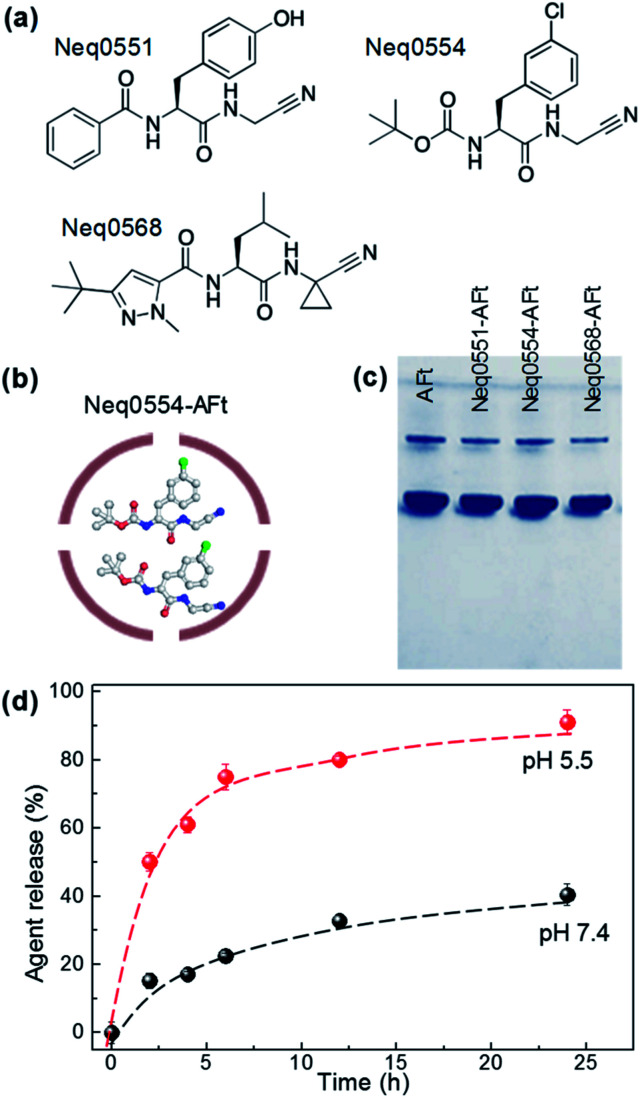
(a) Chemical structures of the test agents. (b) A schematic representation of encapsulated agent, Neq0554-AFt. (c) A photograph of native PAGE of AFt and AFt-encapsulated agents. (d) Drug release profile for Neq0554-AFt at pH 5.5 and pH 7.4 at *T* = 37 °C.

The dipeptidyl nitriles derivatives were synthesized as described^18^ and agents were selected for this study based on their biochemical potential to inhibit cysteine proteases in the nanomolar range. Three reversible covalent cysteine protease inhibitors based on dipeptidyl nitrile were used: Neq0551 ((2*S*)-*N*-(cyanomethyl)-3-(4-hydroxyphenyl)-2-(phenylformamido)propenamide); Neq0554 ((2*S*)–*N*-(cyanomethyl)-2-{[1-methyl-3-(trifluoromethyl)-1*H*-pyrazol-5-yl]formamido}-3-phenylpropanamide) and Neq0568 ((2*S*)-2-[(3-*tert*-butyl-1-methyl-1*H*-pyrazol-5-yl)formamido]-*N*-(cyanomethyl)-4-methylpentanamide) (Fig. 1a). All agents were encapsulated into the AFt cage by passive diffusion through the six hydrophobic channels in the protein cage (Fig. 1b).^28^

For encapsulations, all agents were dissolved in DMSO (10 mM). Horse spleen AFt was prepared with a concentration of ∼5 mM and was dialyzed against sodium acetate buffer (pH 5.5). For encapsulation, 100 μL of agent was added ten times (with intervals of 45 min between additions) to 1 mL of apoferritin under constant mixing at 4 °C. The final molar ratio of AFt : agent was 1 : 500. The encapsulation of test agents was assessed by UV-Vis and the drug concentration was quantified according to the Beer–Lambert law.

The mean numbers of encapsulated Neq0554 and Neq0551 molecules were 105 and 117, respectively, corresponding to encapsulation efficiencies (EE) > 50% and drug loading >10%. For Neq568, the EE was 71% and the mean number of molecules encapsulated was 226 (Table 1).

**Table 1:** Summary of encapsulation efficiency (EE) and drug loading (DL) for cysteine protease inhibitors. Number of molecules of test agent per AFt capsule was determined by UV-vis spectroscopy. All measurements were performed in triplicate and the standard deviation (SD) is provided

	Chemical formula	*N* (per AFt)	EE (%)	DL (%)
Neq0551	C_18_H_17_N_3_O_3_	117 ± 3	55.2 ± 4.6	14.5 ± 4.4
Neq0554	C_17_H_16_F_3_N_5_O_2_	105 ± 2	50.9 ± 10.9	10.1 ± 1.2
Neq0568	C_17_H_27_N_5_O_2_	226 ± 12	71.2 ± 9.9	14.3 ± 6.8

Following encapsulation, AFt retained its structural integrity, as confirmed by native PAGE^29^ and dynamic light scattering (DLS), revealing the AFt band corresponding to MW ∼ 480 kDa (Fig. 1b) and protein cage diameter of ∼13 nm (Fig. 1c), respectively, as expected for AFt.^30^ The value of the zeta-potential measured for AFt alone was −8.6 ± 0.8 meV, and was not changed following encapsulation of test agents (−8.6 ± 0.7 meV for Neq0554-AFt, −8.5 ± 0.5 meV for Neq0551-AFt, −8.9 ± 0.2 meV for Neq0568-AFt). These results confirm that the agents are encapsulated predominantly inside the AFt cavity. We have studied the release rate for all our encapsulated agents at physiologically relevant pH 7.4 and pH 5.5. We observed markedly more rapid release of cargo at pH 5.5 compared to physiological pH 7.4, with corresponding out-diffusion of Neq0554 > 75% and ∼32% respectively after 12 h dialysis (Fig. 1d). This trend was observed for all test agents (Fig. SI-2†). In addition, at pH 5.5, initial fast release was more evident with >50% compound liberated in the first 6 h.

### Biological activity of AFt-Neq test agents

**Fig. 2:**
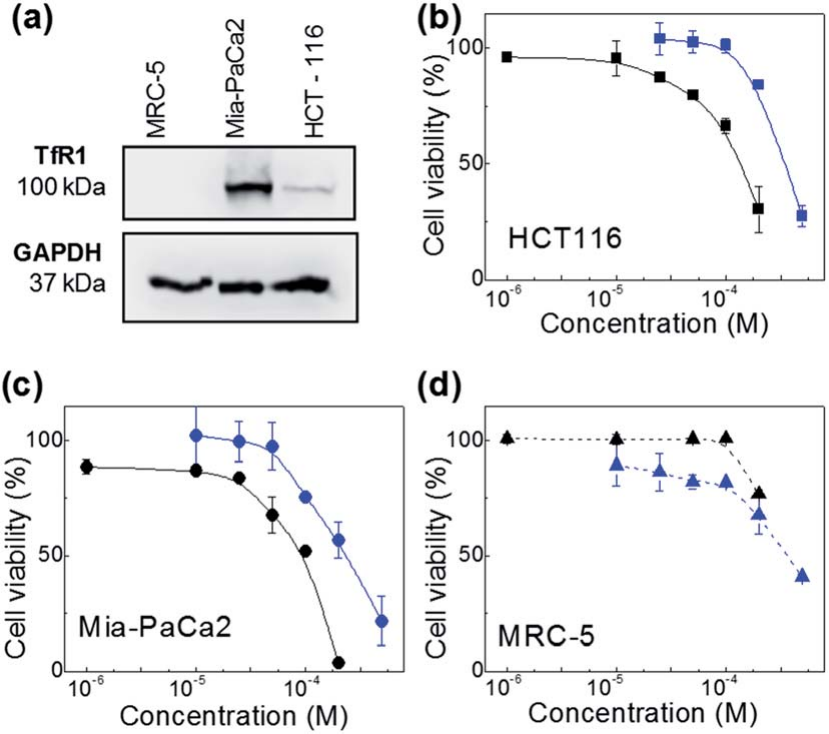
Western blot image revealing expression of transferrin receptor (TfR1) in MiaPaCa-2 and HCT-116 cancer cells (a). Dose response profiles of naked- and AFt-encapsulated Neq0554 in (b) HCT-116, (c) MiaPaCa-2 cancer cells and (d) MRC-5 fibroblasts. Cell viability assays were performed in triplicate (*n* = 4 per trial) and the standard deviation (SD) is shown. Lines are a guide to the eye.

The therapeutic activity of the test agents and their encapsulated formulations was evaluated *in vitro*. For these studies the following cell lines were selected: HCT-116 (CRC) and MiaPaCa-2 (pancreatic) carcinoma cell lines, MRC-5 foetal fibroblasts representative of a non-transformed phenotype. Neq0551, Neq0554 and Neq0568 agents were shown to possess modest growth inhibitory activity against HCT-116 and MiaPaCa-2 carcinoma cells with GI_50_ values ∼400 μM. Selectivity for cancer cells over MRC-5 fibroblasts was unremarkable with maximum cancer selectivity indices achieved for Neq0554 and Neq0568 of 1.75 and ∼2 respectively (GI_50_ MRC-5/GI_50_ MiaPaCa-2 or HCT-116). AFt-encapsulated test agents showed greater growth inhibitory activity in cancer cells, compared to the naked compounds. In particular, following 72 h exposure to carcinoma cells, >2.5-fold enhanced Neq0554 potency was achieved for encapsulated agent; against MiaPaCa-2 cells, GI_50_ value < 80 μM for AFt-Neq0554 (Fig. 2). AFt-encapsulation of Neq0568 reduced the GI_50_ value >3-fold in MiaPaCa-2 cells (Table 2). Importantly, selectivity for cancer cells became more pronounced for encapsulated agents with GI_50_ values of >200 μM in non-tumorigenic MRC-5 fibroblasts.

**Table 2:** GI_50_ values for both naked and AFt-encapsulated drugs tested against the cancer and normal cell lines

Test agent	Mean GI_50_ values ± SD (μM)		
	HCT-116	MiaPaCa-2	MRC-5
Neq0551	>500	>500	>500
Neq0551-AFt	>200	162.2(±5.7)	>200
Neq0554	358.6(±7.7)	230.7(±9.1)	404.0(±9.7)
Neq0554-AFt	131.0(±5.2)	79.5(±10.7)	>200
Neq0568	231.1(±7.8)	393.0(±8.1)	>500
Neq0568-AFt	168.1(±6.5)	125.1(±9.8)	>200

AFt is internalized by cells by TfR1-mediated endocytosis,^25^ hence western blot was performed to investigate cellular TfR-1 levels. TfR1 protein expression was detected in lysates prepared from HCT-116 and MiaPaCa-2 cells; in contrast, TfR1 levels were undetectable in lysates of non-tumorigenic fibroblasts, inferring disparity in TfR1 expression between cancer and non-cancer cells (Fig. 2d). Indeed, rapid cell division increases cellular iron demand and enhanced TfR1 expression is detected in cancer cells.^25^ Preferential expression of TfR1 by actively dividing cells, including normal fibroblasts, was reported >30 years ago,^31^ as transferrin is required for cell proliferation in culture.

**Fig. 3:**
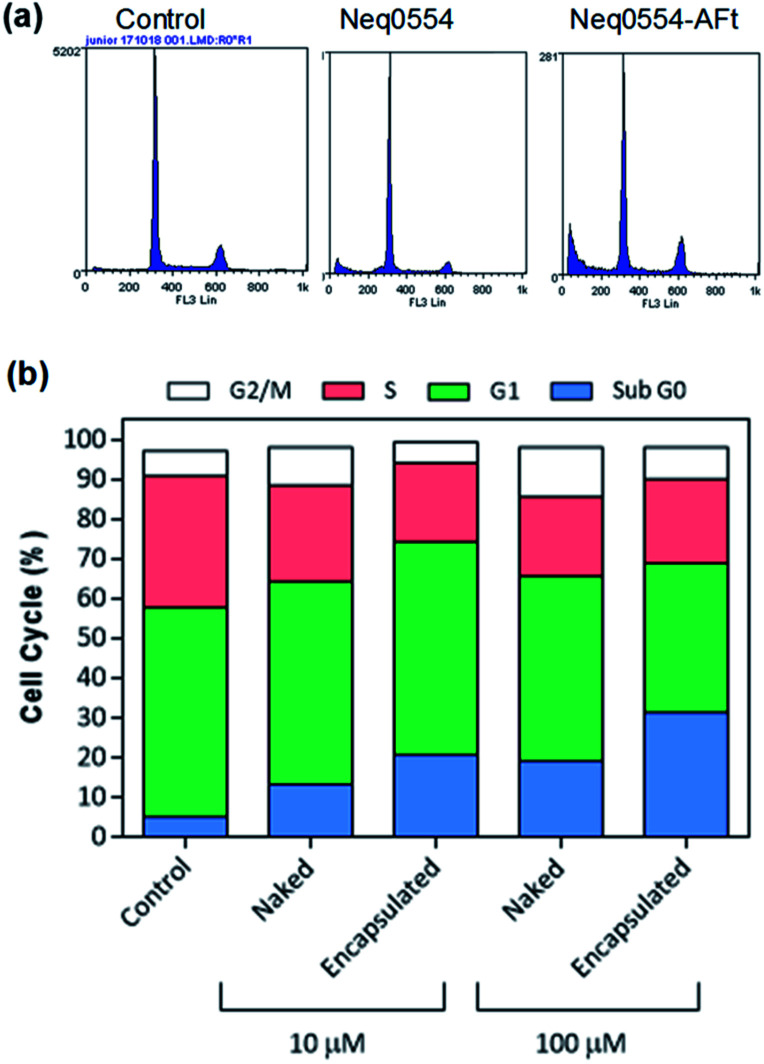
(a) Representative cell cycle profiles of HCT-116 cells and (b) histogram of the cell population distribution for control cells and following treatment with naked and encapsulated Neq0554. The standard deviation was calculated and was found to be <5%.

To better understand the mechanism of carcinoma cell growth inhibition caused by AFt-Neq0554 compared to naked agent, cell cycle was examined by flow cytometry. Following 48 h treatment, MiaPaCa-2 and HCT-116 cells were gently permeabilized and cellular DNA was stained with propidium iodide (PI). Although changes in G_1_, G_2_ and M phase-events were modest, the population of cells in S-phase was reduced by ∼15% in HCT 116 cells following treatment with AFt encapsulated agent AFt-Neq0554 (10 μM). These results suggest reduced DNA replication and cytostasis/quiescence. Also, following exposure of cancer cells to both naked and encapsulated agents, small but significant pre G1 phase populations were observed, indicating apoptosis. Representative cell cycle profiles for HCT 116 (Fig. 3) show 14% and 26% of pre-G_1_ events following exposure to 10 μM and 100 μM AFt-encapsulated Neq 554, respectively.

Clonogenic assays were performed to explore further the putative cytotoxicity caused by AFt-Neq0554, examining whether cells surviving exposure to the test agents retain the ability to form colonies. The clonogenic survival of HCT 116 cells was marginally inhibited (22% and 28%) by 24 h exposure to 100 μM naked and AFt-encapsulated Neq0554, respectively. In contrast, MiaPaCa-2 colony formation was dramatically impeded (>60%) by naked and AFt-encapsulated Neq0554 (100 μM). In both cell lines, AFt-encapsulated Neq554 inhibited colony formation to a greater extent than naked agent, likely due to enhanced cellular uptake. That colony formation persisted, strongly indicates a cytostatic response to treatment, temporary quiescence dependent upon presence of the compound.

### Cathepsin L inhibition analysis by confocal microscopy

**Fig. 4:**
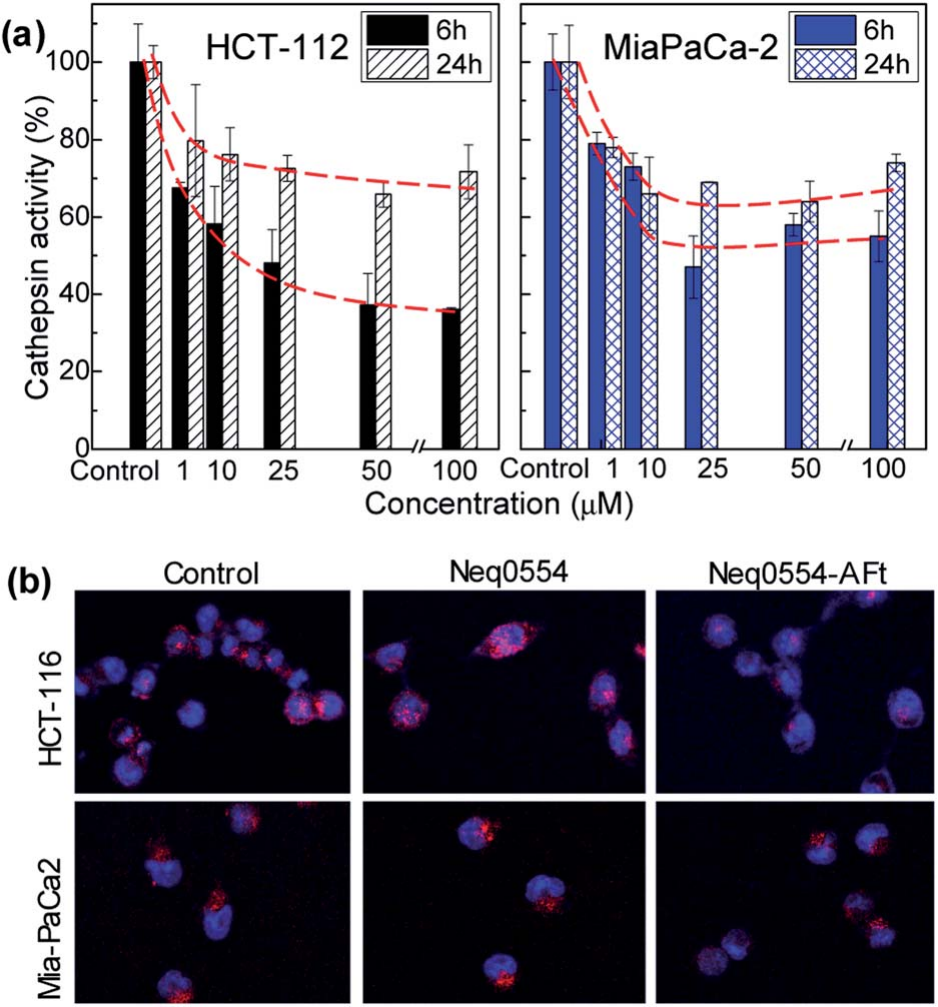
Cathepsin L *in vitro* activity in HCT 116 and MiaPaCa-2 cells following 6 h or 24 h exposure to AFt-Neq554. Data were generated following quantification of confocal microscopy images and represent mean ± SD ≥ 3 independent trials (a). Confocal images of HCT 116 and MiaPaCa-2 cells after 6 h incubation of 50 μM naked and AFt-encapsulated Neq554, for comparison, images of untreated control cells are also shown (b). All images are 100 μm wide.

Cells were exposed to different concentrations of naked and encapsulated Neq0554 for 6 and 24 h to examine cathepsin L inhibition inside live cells. In both cancer cell lines, total cathepsin L activity was more effectively inhibited following exposure to AFt-encapsulated agent, compared to naked Neq0554. AFt alone had no effect on cathepsin L activity. At treatment concentrations of Neq554 above 25 μM, inhibition of cathepsin L by AFt-encapsulated agent was >50% after 6 h exposure (Fig. 4). Although some recovery of activity was observed after 24 h treatment, cathepsin L inhibition >20% did persist. This trend was also observed in MiaPaCa-2 cells. Of note was the observation that after 24 h exposure of cells to naked Neq0554, no inhibition of cathepsin L was detected in either cell line, activity equivalent to untreated control cells was observed (not shown) strongly inferring that AFt-encapsulation prolonged cathepsin L inhibition, potentially by retaining intracellular/lysosomal Neq554 for a greater period of time.

All inhibitors used here were designed based on a dipeptidyl nitrile structure as prototype.^19^ Using biochemical analysis, we were able to measure the affinity of these drugs to their molecular target: cathepsin L. The apparent inhibition constant *K*_i_′ was determined by non-linear regression to be 7.10 ± 0.04, 7.90 ± 0.04 and 8.10 ± 0.03 for Neq0551, Neq0554 and Neq0568, respectively. We observed that all compounds were potent inhibitors of cathepsin L activity based on an *in vitro* assay, with *K*_i_′ lower than 10 nM. Biochemical affinity has been corroborated with observations of cellular cathepsin L inhibitory activity in real time.

## Discussion

The loading of the cysteine protease inhibitor in AFt nanocages was performed by passive diffusion, where agents enter the cage *via* channels in the AFt capsule. Following encapsulation, the samples are purified to remove non-encapsulated drug molecules. We assessed the morphology of encapsulated agents by DLS and native PAGE, confirming that the external diameter and surface charge of the AFt remains unchanged following encapsulation. We also note, that enhanced intracellular internalisation of AFt-encapsulated agent is strongly supported by our *in vitro* studies and confocal microscopy imaging of cathepsin L inhibition. These observations strongly suggest that the protein remains unchanged following the encapsulation process. Hence, we conclude that all molecules detected in our samples are in the inner cavity of the protein.

The covalent reversible cysteine protease inhibitors used in this work were reported previously as experimental putative anticancer agents,^18,19^ however their selectivity to cancer cell lines needed to be addressed to reduce potential toxicities and side effects. All compounds are dipeptidyl nitrile derivatives (Fig. 1a). The cysteine protease inhibition occurs by interaction of nitrile group with the sub sites present in the enzyme structure, promoting the attack of the nitrile group by the nucleophilic Cys25 in the catalytic pocket of the cysteine protease.

Based on previous results from our group, the nanomolar inhibition of the cysteine proteases did not lead to relevant cytotoxic activity at 100 μM in cell-based assays against the *Leishmania* spp. parasites 30 or pancreatic carcinoma cells (MiaPaCa-2).^30,32^ Therefore, AFt encapsulation was used to enhance the cytotoxic potential against cancer cells. Since the test agents are hydrophobic (Fig. 1a), we envisage that encapsulation takes place *via* passive diffusion through the hydrophobic channels in the protein cage (Fig. 1b) in which EE > 50% was achieved for all compounds. Also, the surface charge of the AFt nanocapsules was not affected following the encapsulation procedure, suggesting that the molecules are incorporated within the cavity and are not attached to the protein capsules' exterior (Fig. 1c), hence the cellular recognition and uptake of AFt is not expected to be affected by the presence of internalized agent.

An additional benefit of the AFt formulation arises from the pH sensitivity of the protein capsule,^33^ which can be employed for favourable pH controlled drug release under specific conditions. Our release studies confirmed that at reduced pH values associated with increased size of the AFt channels (pH 5.5),^34,35^ the release of the agent is enhanced by a factor of 4 compared to pH 7.4 (Fig. 1d and ESI, Fig. S2†), similar to that demonstrated for doxorubicin.^35^ In contrast, markedly reduced drug release was obtained at physiological pH (Fig. 1d). Previous reports also indicated long-term retention of encapsulated agents at pH 7.^34,35^ Our results confirm that the drug encapsulated within AFt nanocages will be preferentially released in acid environments such as those within the cancer microenvironment and specifically, intracellular lysosomes.^36^

We posit that AFt provides a biocompatible carrier, indeed, no cytotoxic activity of AFt alone was observed in any of the studied cell lines (Fig. SI-2†), consistent with work reported previously.^37^ In addition, encapsulation of biomolecules into AFt nanocages has excellent potential in terms of enhanced drug accumulation, cellular uptake and biological activity.^38^

AFt is preferentially internalized by cells following TfR1-mediated endocytosis,^25^ hence AFt encapsulation of active agent provides a tool for their selective uptake by cells expressing TfR1, hence decreasing possible side effects.^34^ Cellular TfR-1 protein was detected in lysates of HCT-116 and MiaPaCa-2 cancer cells, and was below detectable levels in non-tumorigenic fibroblast MRC-5 cells (Fig. 2a). This difference in TfR1 expression is a likely cause of the improved potency against cancer cells of all test agents after AFt-encapsulation. Also, selectivity indices for Neq compounds are clearly enhanced between cancer and non-cancer cells, as evident from the dose response curves (Fig. 2b–d). Following exposure of cancer cells to both naked and encapsulated Neq0554, increased pre-G1 phase populations were observed (Fig. 3), indicating apoptosis.^39,40^

Cell survival and clonogenic expansion are fundamental to cancer development and metastases.^41,42^ Clonogenic assays were therefore performed to explore further putative cytotoxicity imparted by AFt-Neq0554, examining whether cells surviving exposure to the test agents retain the ability to form colonies. In both studied cancer cell lines, AFt facilitated increase of cellular uptake of the agent led to inhibition of colony formation to a greater extent than naked agent. Clonogenic survival was significantly lower in MiaPaCa-2 compared to HCT-116 cells. In addition, colonies of reduced size were observed following treatment of cells with AFt-encapsulated agents. Indeed, cysteine protease inhibition is expected to decrease cell migration and invasion,^8^ key cancer hallmarks essential for metastasis.

Inhibitors of proteases have shown *in vitro* and *in vivo* anti-cancer activity, promoting beneficial effects for the treatment of tumours.^43^ Herein, we demonstrate that cathepsin L inhibition was markedly enhanced in both cell lines after exposure to AFt-Neq0554. These observations corroborate a role for TfR1-mediated uptake of AFt-encapsulated agents and sustained release of cargo in acidic lysosomes, indicating the significance of AFt nanocage-encapsulation to potentiate treatment efficacy by exploitation of cancer cell upregulation of TfR1. It is noteworthy that in MiaPaCa-2 cells, where cathepsin L inhibition persisted for 24 h, clonogenic survival, following 24 h exposure of cells to AFt-Neq0554 was <40%, thus inferring that the cysteine protease inhibition is intensified by encapsulation of the inhibitors in AFt, which traffics cargo directly to the lysosomes, where these enzymes predominate.^44^ Cathepsin L inhibition was demonstrated previously using small molecule inhibitors,^7^ antisense RNA^45^ and (si)RNA.^46^ The anticancer activity of AFt-encapsulated dipeptidyl nitrile based inhibitors is comparable to the activity of other small molecule inhibitors,^7^ and offers additional benefit of pH induced release and selective uptake. Inhibition of cathepsin L achieved in this work is of particular importance for pancreatic cancer treatment, where cathepsin L is considered to be an independent prognostic marker,^47^ and its inhibition could be used to reduce cancer invasion and tumor growth, and merits further investigations *in vivo*.

## Conclusions

In conclusion, we have developed a robust method for encapsulation of novel Neq cathepsin L inhibitors into AFt nanocages: >100 Neq0554 molecules were encapsulated per AFt cage achieving >50% EE. The integrity of AFt nanocages was preserved as evidenced by DLS and native PAGE. Encapsulated Neq0554 demonstrated enhanced anticancer activity *in vitro* compared to naked agent (3-fold), revealing cancer cell line-selectivity (2.5-fold), possible consequence of enhanced TfR-1-mediated endocytosis of AFt-Neq0554. Increased and more persistent inhibition of cathepsin L in treated cancer cells was observed following AFt-encapsulation of Neq0554, consistent with cellular retention and sustained release of Neq0554 in acidic cytosolic compartments. Release of all Neq cathepsin L inhibitors, determined under physiological conditions demonstrated more rapid liberation from AFt capsules in acidic environments analogous to those found in lysosomes. We conclude that AFt-mediated delivery of novel cathepsin L inhibitors is worthy of further pursuit as a putative anticancer strategy.

## Conflicts of interest

All the authors confirm there are no conflicts to declare.

## Supplementary Material

RA-009-C9RA07161J-s001
