# Supplementary material for: Apoferritin encapsulation of cysteine protease inhibitors for cathepsin L inhibition in cancer cells
Source: RSC Adv. 2019 Nov 11;9(63):36699–706. doi: 10.1039/c9ra07161j (PMC9075514; doi:10.1039/c9ra07161j)
Supplement: RA-009-C9RA07161J-s001 [file RA-009-C9RA07161J-s001.pdf]

Electronic Supplementary Material (ESI) for RSC Advances.
This journal is © The Royal Society of Chemistry 2019

SUPPLEMENTARY INFORMATION

Apoferritin encapsulation of cysteine protease inhibitors for cathepsin L inhibition in cancer cells

José C. Quilles Junior, Fernanda dos Reis Rocho Carlos, A. Montanari, Andrei Leitão, Viviane W. Mignone Maria Augusta Arrudac, Lyudmila Turyanska, Tracey D. Bradshaw

SI1. Characterization of apoferritin encapsulated agents

Encapsulation of agents into the AFt cavity does not affect the morphological properties of the protein. Apoferritin retains its size and surface charge. Results of DLS measurements and a summary of the hydrodynamic sizes observed are shown in the Figure S1. Release of the agents was assessed at pH 7 and 5.5 and the release profiles are shown in the Figure S2.

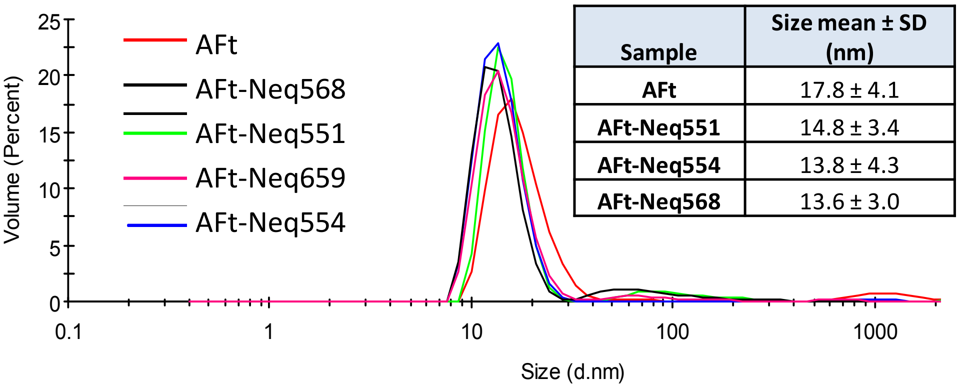

Sample	Size mean ± SD (nm)
**AFt**	17.8 ± 4.1
**AFt-Neq551**	14.8 ± 3.4
**AFt-Neq554**	13.8 ± 4.3
**AFt-Neq568**	13.6 ± 3.0

Figure S1. Hydrodynamic size of AFt and AFt-encapsulated agents measured by DLS. Summary of the sizes observed is presented in the Table in the inset.

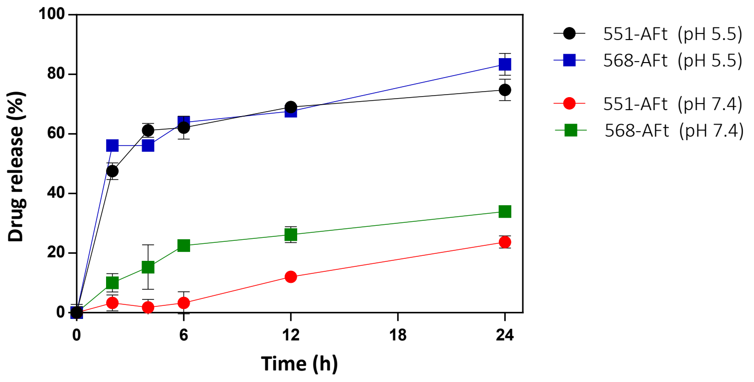

Figure S2. Drug release of encapsulated Neq0551 and Neq0568 compounds incubated for 24 hours at pH 5.5 and pH 7.4. The assay was performed in triplicate and the values represent the average with the SD (%).

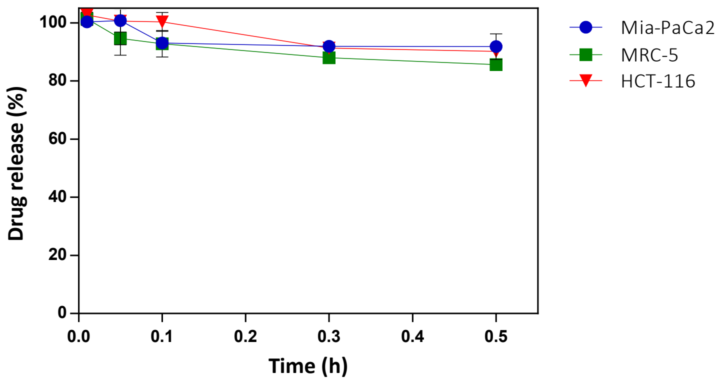

Figure S3. Biocompatibility assay using different concentrations of AFt incubated with the cell lines for 72 hours. The assay was performed in triplicate and the values represent the average with the SD (%).
